# Mucosal coronally positioned flap technique for management of excessive gingival display

**DOI:** 10.11604/pamj.2020.36.235.22597

**Published:** 2020-07-30

**Authors:** Adel Bouguezzi, Ouiam Hiba Boudour, Sameh Sioud, Hajer Hentati, Jamil Selmi

**Affiliations:** 1 Faculty of Dental Medicine, University of Monastir, Monastir, Tunisia,; 2 Dental Clinic of Monastir, Department of Medicine and Oral Surgery, Oral Health and Oro-Facial Rehabilitation Laboratory Research (LR12ES11), 5019, Monastir, Tunisia

**Keywords:** Gummy smile, lip repositioning, mucosal coronally flap

## Abstract

Improvement of smile esthetics is a major goal of modern dentistry. Various treatment modalities have been proposed to correct excessive gingival display, depending on intraoral or extraoral etiologies. This report aimed to document the use of mucosal coronally positioned flap called surgical lip repositioning technique for the management of a gummy smile associated with vertical maxillary excess. The procedure restricts the muscle pull of the elevator lip muscles by shortening the vestibule, thus reducing the gingival display when smiling. Rapid surgical healing with minimal postoperative sequelae was observed. The follow up examinations showed esthetic satisfaction up to 6 months postoperatively, at the end of one year a partial relapse was observed. Although the short-term stable results of lip repositioning surgery appear satisfying postoperatively, its utility as a long-term treatment option remains questionable. More studies with larger sample size and long-term follow-up are necessary to establish the level of scientific evidence of this procedure.

## Introduction

Recently, the demand for esthetics has significantly increased, driven by increased patient awareness and the search for an ideal smile. Creating the perfect smile is an intricate process that requires a multidisciplinary approach, with careful consideration of the lips and the gingival outline. Patients desire to look good not only in a static pose but also during dynamic facial expression. Excessive gingival display is a clinical finding with many etiologies and may include extraoral or intraoral components. Some extraoral causes of a gummy smile are vertical maxillary excess (VME), hypermobile upper lip (HUL), or a short upper lip. A visual diagnosis of VME is made when the lower third of the face is longer than the remaining thirds; cephalometric analysis can be used as an additional aid. The best orthodontically treated patients may not be satisfied by the treatment if soft tissue problem is not corrected. Treatment for the most extraoral or intraoral cause of gummy smile, except for a short or hypermobile lip has been well documented. Various surgical and nonsurgical modalities have been described in the treatment of gummy smile which includes Lefort I osteotomy, crown lengthening procedures, maxillary incisor intrusions, micro implants, headgears, self-curing silicone implant injected at anterior nasal spine (ANS) with myectomy, and partial resection of levator labii superioris with muscle repositioning. However, these procedures do not help in reducing the hyperactivity of the muscles and therefore, nonsurgical treatment may be a desirable option.

## Patient and observation

**Figure 1:**
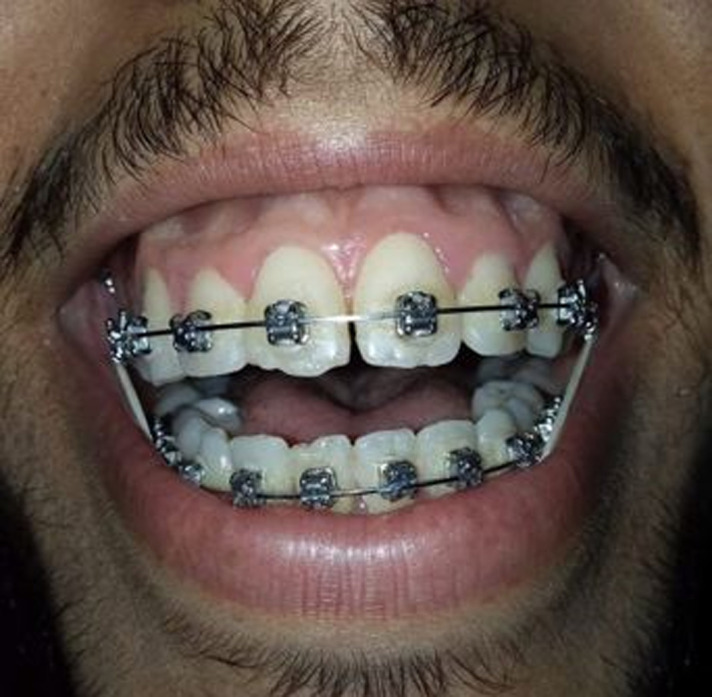
excessive gingival display preoperatively

**Figure 2:**
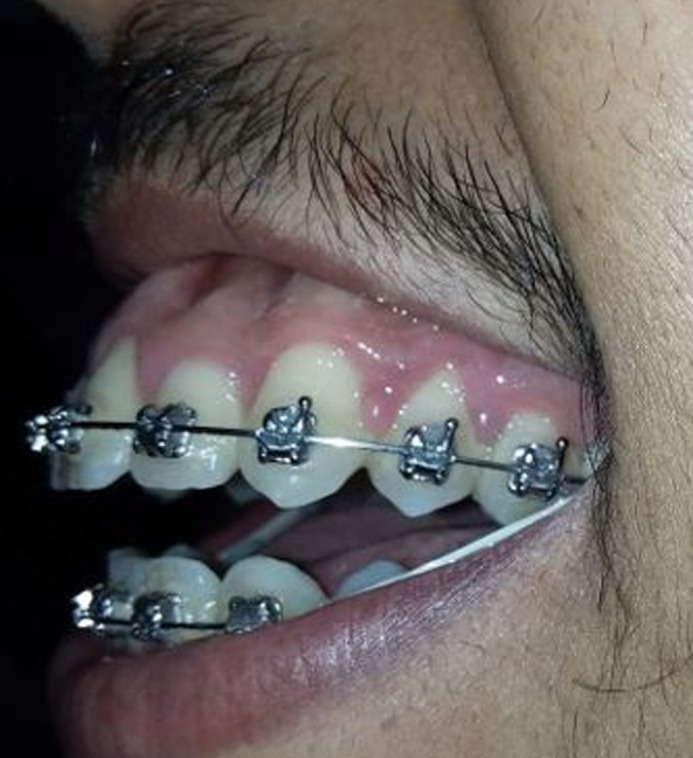
close-up smile, left lateral view

A 24-year-old man reported to the department of medicine and oral surgery with the chief complaint of excessive gingival display during smile. There were no significant medical or family history and the patient presented with no medical conditions that could contradict the surgical procedure. On clinical examination, the extra orally face was bilaterally symmetrical with incompetent lips. Intraorally a good amount of attached gingiva was seen, and the patient was diagnosed with a case of “gummy smile.” However, when the patient was asked to smile, his teeth were visible from maxillary right first molar to maxillary left first premolar with 7-8 mm gingival display and the maxillary anterior teeth had normal anatomic proportions (Figure 1, Figure 2). Patient was given the option of orthognathic surgery to correct his VME or lip repositioning procedure (LRP) to reduce his gummy smile and improve esthetics. He elected LRP to address the gummy smile and avoid higher morbidity and costs associated with orthognathic surgery. The patient´s expectations were clarified, and a realistic outcome was presented, including the possibility of full or partial relapse. Written informed consent was obtained after an explanation of the risks, potential benefits, and treatment alternatives. The aim of the technique lip repositioning is a surgical way to correct gummy smile by limiting the retraction of the elevator smile muscles (orbicularis oris, levator anguli oris, levator labii superioris, zygomaticus minor).

**Figure 3:**
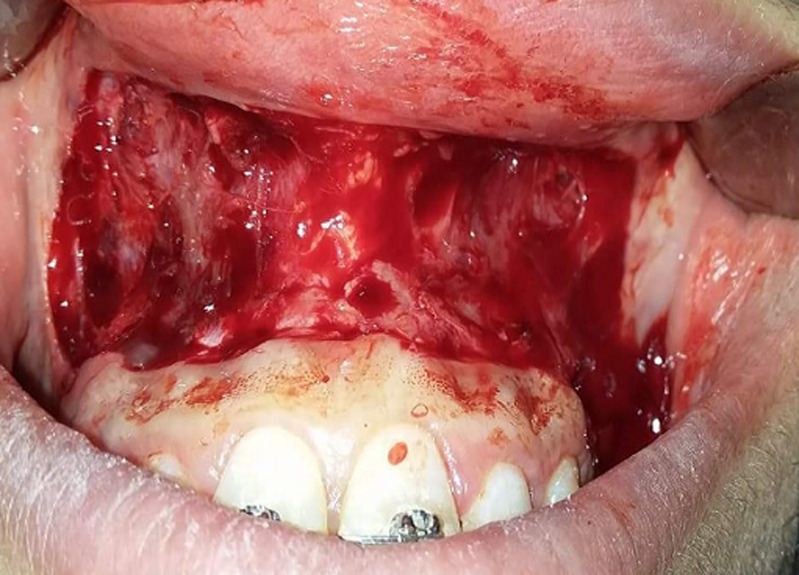
the tissue was removed via a partial thickness dissection with tissue removal from first molar to first molar

**Figure 4:**
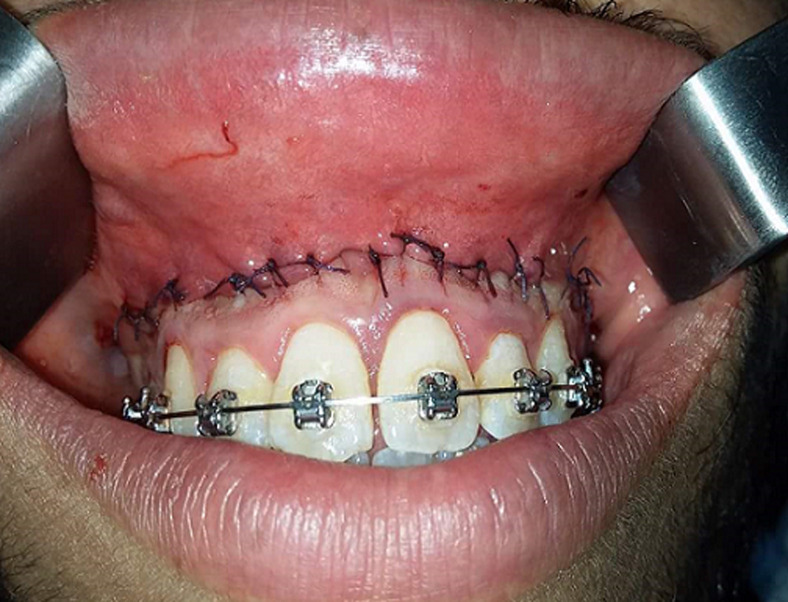
final suturing for stabilization of the new mucosal margin to the gingiva

**Figure 5:**
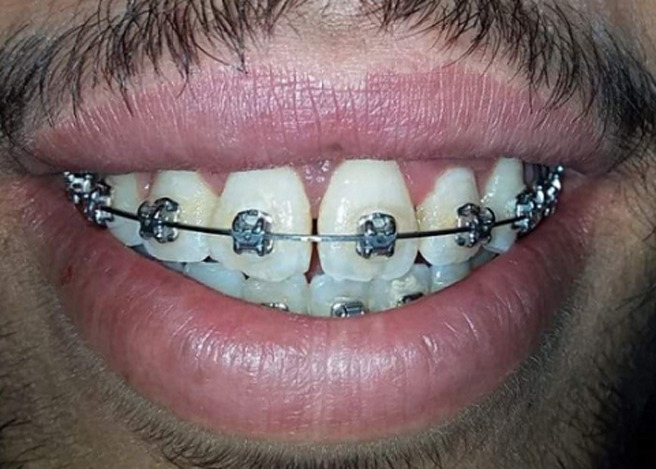
immediately postoperatively view with moderate smile

Local anesthesia (Mepivacaine 2% with adrenaline 1: 80,000) was administered at the vestibular mucosa and lip from the maxillary right to left first molar. The incision outline was marked with an indelible pencil on the dried tissue using mucogingival junction and base of the vestibule as reference lines connected at mesial line angles of the right and left maxillary molars to create an elliptical outlineas. Incisions were made in the surgical area, and both superior and inferior partial thickness flap was raised from maxillary right second premolar to maxillary left second premolar. A partial thickness incision was made between these two lines, and epithelial band approximately 10-12 mm wide was excised leaving underlying connective tissue exposed (Figure 3). Care was taken to avoid damage to any minor salivary glands in the submucosa and tissue tags were removed. The mucosal flap was advanced and multiple interrupted sutures at 1 mm were taken at the midline and other locations along the borders of the incision mucogingival junction using 3-0 vicryl sutures (Figure 4). No periodontal dressing was placed. Immediately postoperatively significant improvement in the patient facial and smile was observed (Figure 5).

**Figure 6:**
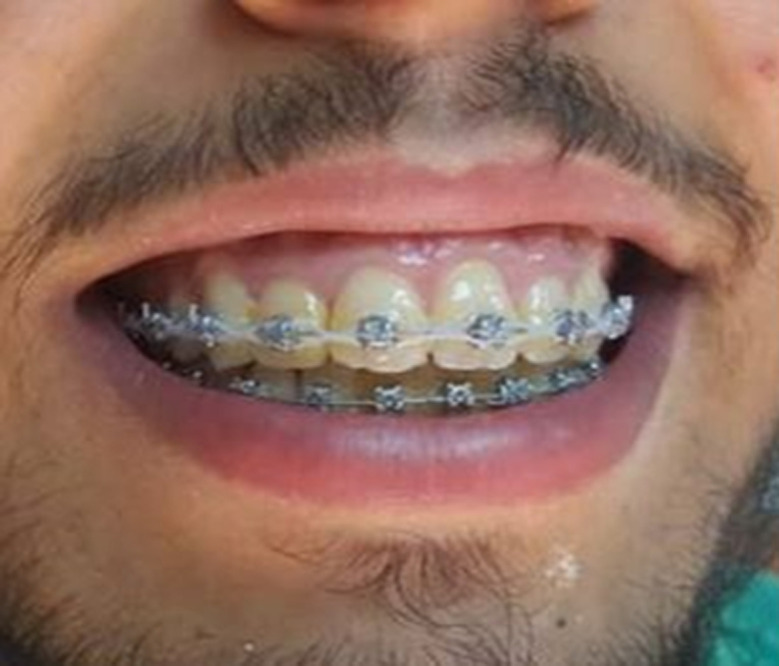
postoperatively clinical view at 12-month follow-up

Post-operative instructions included soft diet, limited facial movements, no brushing around the surgical site for 14 days and placing ice packs over the upper lip. The patient was instructed to rinse gently with 0.2% chlorhexidine gluconate twice daily for 2 weeks. Post-operative Amoxicillin 500 mg and Ibuprofen 400 mg B.D for 5 days were prescribed. Post-operative healing occurred, and the patient reported minimal post-operative bruising, or extraoral swelling and slight pain when smiling for 1 week after surgery. Sutures were removed 2 weeks later. The suture line healed in the form of a scar that was concealed in the upper lip mucosa and not visible when the patient smiled. This esthetic procedure is safe and has minimal side-effects. The patient was recalled after every 3 months for follow-up. Gingival display at baseline was 7-8 mm which changed drastically at 3 and 6 months postoperatively. At 3 months and at 6 months gingival display was 3 mm. There was no difference in gingival display between 3 and 6 months. However, a partial relapse was observed after 12 months (Figure 6).

## Discussion

Lip repositioning was first described in the literature of plastic surgery in 1973 by Rubinstein and Kostianovsky [1], which was advocated again by Litton and Fournier [2] for correction of excessive gingival display in the presence of short upper lip. Patient who have a high lip line exposes a broad zone of gingival tissue and may often express concern about their 'gummy smile' that can be a source of embarrassment for some patients. When a person smiles, the entire crown of the maxillary central incisors and 1 mm of pink attached gingiva will be evident. Greater amount of exposed gingiva (2-3 mm) can be cosmetically acceptable as long as the gingiva is not unduly conspicuous such as “Gummy smile” appearance, where more than 3 mm of gingiva is displayed during a relaxed smile. This correction of esthetics should be done under the boundaries of “cosmetic zone.” In an esthetic evaluation of dentogingival complex, the midline of the face, the position of the incisal edges and the gingival line are important landmarks [3]. The form of the lips and the position of the lips during speech and smiling cannot be easily changed, but the dentist may, if necessary, modify/control the form of the teeth and interdental papillae as well as the position of the gingival margins and the incisal edges of the teeth along with repositioning of the lip. In other words, it is possible by a combination of periodontal and prosthetic treatment measures to improve dentofacial esthetics [4].

This report aimed to document lip repositioning technique to decrease the amount of gingival display in patients with gummy smile. The results showed esthetic satisfaction up to 6 months postoperatively, at the end of one year a partial relapse was observed. The results showed that the employed surgical procedure successfully reduced the gingival display with low morbidity. The procedure is safe and has minimum side effects [5]. Reports in the literature have shown minimal post-operative bruising, discomfort and swelling. Mucocele formation has been the most severe reported complication [6]. Lip repositioning procedure began as a plastic surgical treatment and ever since variations have been reported [7]. The original technique did not include severing the muscle attachment after flap reflection [1,5]. However, some authors suggested performing myectomies to detach smile muscle attachment to prevent relapse [8,9]. Another method to prevent reattachment of smile elevator muscles is the placement of spacer between elevator muscles of lip and anterior nasal spine thereby preventing superior displacement of repositioned lip [10].

The contraindications for this technique include the presence of a minimal zone of attached gingiva, which can create difficulties in flap design, stabilization and suturing. Another contraindication is several vertical maxillary excess (VME). Degree II VME has gingival and mucosal display of 4 to 8 mm. In the other hand, in degree III VME more than 8 mm of soft tissue are seen. In both cases, an interdisciplinary approach is required [11]. In order to determine other factors that are not related with the hyperfunction of the upper lip elevator muscle, certain characteristics must be taken in account. Facial proportions must be symmetric in the three horizontal thirds, without identification of higher proportion of the inferior third, which could characterize an excessive maxillary vertical growth. Another factor to be evaluated is the distance between the gingival margin and CEJ, which ideally is < 1.5 mm. Distances greater than 1.5 mm indicate an excessive gingival tissue covering the tooth crown, typical in altered passive eruption. Finally, the crown length-height relation must be evaluated. The maxillary central incisor´s width must be about 80% of its length, with an accepted variation between 65% and 85%, and the maxillary lateral incisors about 70% [12].

Surgical crown lengthening, orthodontic treatment, orthognathic surgery, Botox injection and surgical lip repositioning are the different treatment modalities that has been used in the treatment of excessive gingival display or gummy smile [13,14]. Surgical crown lengthening is best option when there is gingival hypertrophy or short clinical crown due to altered passive eruption. However, surgical crown lengthening in maxillary overgrowth case can lead into unaesthetic long crowns in the maxillary anterior. Orthognathic surgery is the treatment of choice for the maxillary overgrowth. However, cost, invasiveness and the post-operative morbidity of this technique cannot be justified with the treatment outcomes in minor vertical discrepancy. Botox injection in management of the gummy smile has brought the new horizon of non-surgical and more conservative treatment option in the case of esophagogastroduodenoscopy (EGD) [15]. However, cases treated with Botox injection has the maximum relapse tendency within 3 to 6 months [2,16]. Surgical management of the EGD due to short and hyperactive upper lip includes lip elongation associated with rhinoplasty, detachment of lip muscles, myotomy, and lip repositioning [17].

**Figure 7:**
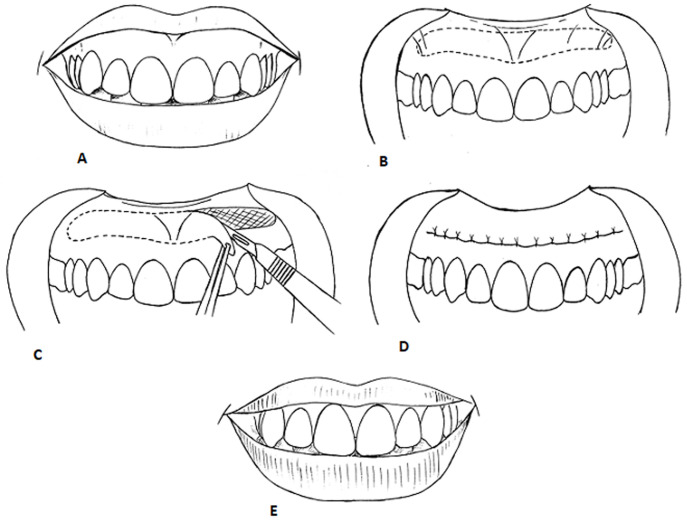
schematic illustration of the lip repositioning technique; (A) preoperative image of the dynamic smile; (B) borders of the surgical excision; (C) epithelial excision and exposed connective tissue; (D) suturing; (E) final situation

Patient undergoing lip repositioning surgery should be healthy, with no periodontal disease. It should be performed under infiltration of adequate amount of local anesthesia from maxillary right first molar to left first molar. Then the incision area is marked by sterile pencil on dry mucosa. First incision should be given along the mucogingival junction and second incision will be given almost parallel to the first incision at labial mucosa about 10- 12 mm away from the mucogingival junction. Two incisions will be connected at the first molars of either side with elliptical outline [16]. The epithelium from the incision outline should be removed by split thickness flap with the exposure of connective tissue bed. Then the two-incision line should be approximated by either interrupted or continuous suture [18] (Figure 7). There is a modified technique in which the maxillary labial frenulum is maintained and two mucosal strips, one at each side of the frenulum, are removed [12]. Leaving the frenulum intact helps maintaining the position of the labial midline, prevents changes in lip symmetry and decreases the morbidity associated with the procedure, but limits the possibility of correcting EGD in the region of the maxillary central incisors [19]. For this reason, in the present cases not to maintain the frenulum was decided. This case report shows that although the results of lip repositioning surgery appear stable for up to 6 months postoperatively, its utility as a long-term treatment option remains questionable. More studies with larger sample size and long-term follow-up are necessary to establish the level of scientific evidence of this procedure.

## Conclusion

The innovative surgical approach discussed in this article is proposed for patients with a gummy smile who have very high esthetic expectations. This technique was designed to be shorter, less aggressive and to have fewer postoperative complications compared to orthognathic surgery. The study and the careful evaluation of the etiology and degree of gummy smile severity are determinant in the selection of the treatment. The etiology of the gummy smile can be multifactorial, however there are cases in which it is possible to identify an isolated etiological factor. The patient has the final word in the therapeutic option, so we must explain to the patient clearly all the appropriate treatment options for his case, exposing all the advantages and disadvantages of each therapeutic approach.

## References

[1] Rubinstein AM, Kostianovsky AS (1973). Cirugia astetica de la malformacion de la sonrisa. Pren Med Argent.

[2] Litton C, Fournier P (1979). Simple surgical correction of the gummy smile. Plast Reconstr Surg.

[3] Sheth T, Shah S, Shah M, Shah E (2013). Lip reposition surgery: a new call in periodontics. Contemp Clin Dent.

[4] Gupta KK, Srivastava A, Singhal R, Srivastava S (2010). An innovative cosmetic technique called lip repositioning. J Indian Soc Periodontol.

[5] Kamer FM (1979). "How do I do it"- Plastic surgery, practical suggestions on facial plastic surgery, smile surgery. Laryngoscope.

[6] Rosenblatt A, Simon Z (2006). Lip repositioning for reduction of excessive gingival display: A clinical report. Int J Periodontics Restorative Dent.

[7] Simon Z, Rosemblatt A, Dorfmann W (2007). Eliminating a gummy smile with surgical lip repositioning. J Cosmetic Dent.

[8] Cachay-Velasquez H (1992). Rhinoplasty and facial expression. Ann Plast Surg.

[9] Miskinyar SA (1983). A new method for correction of gummy smile. Plast Reconstr Surg.

[10] Ellenbogen R, Swara N (1984). The improvement of gummy smile using the implant spacer technique. Ann Plast Surg.

[11] Humayun N, Kolhatkar S, Souiyas J, Bhola M (2010). Mucosal coronally positioned flap for the management of excessive gingival display in the presence of hypermobility of the upper lip and vertical maxillary excess: a case report. J Periodontol.

[12] Ribeiro-Júnior NV, Campos TV, Rodrigues JG, Martins TM, Silva CO (2013). Treatment of excessive gingival display using a modified lip repositioning technique. Int J Period Restorative Dent.

[13] Jankovic J, Brin MF (1997). Botulinum toxin: historical perspective and potential new indications. Muscle Nerve Suppl.

[14] Polo M (2005). Botulinum toxin type A in the treatment of excessive gingival display. Am J Orthod Dentofacial Orthop.

[15] Ezquerra F, Berrazueata MJ, Ruiz-Capillas A, Arregui JS (1999). New approach to the gummy smile. Plast Reconstruct Surg.

[16] Ishida LH, Ishida LC, Ishida J (2010). Myotomy of the levator labii superioris muscle and lip repositioning: a combined approach for the correction of gummy smile. Plast Reconstruct Surg.

[17] Silva CO, Ribeiro-Junior NV, Campos TV (2013). Excessive gingival display: Treatment by a modified lip repositioning technique. J Clin Periodontol.

[18] Simon Z, Rosenblatt A, Dorfman W (2007). Eliminating gummy smile with surgical lip repositioning. J Cosmet Dent.

[19] Gabrić Pandurić D, Blasković M, Brozović J, Sušić M (2014). Surgical treatment of excessive gingival display using lip repositioning technique and laser gingivectomy as an alternative to orthognathic surgery. J Oral Maxillofac Surg.

